# A Novel Approach to Staging and Detection of Colorectal Cancer in Early Stages

**DOI:** 10.3390/jcm12103530

**Published:** 2023-05-17

**Authors:** Monika Zajkowska, Barbara Mroczko

**Affiliations:** 1 Department of Neurodegeneration Diagnostics, Medical University of Bialystok, 15-269 Bialystok, Poland; 2 Department of Biochemical Diagnostics, Medical University of Bialystok, 15-269 Bialystok, Poland

**Keywords:** CRC, chemokines, biomarker, cancer detection, index, ratio

## Abstract

Colorectal cancer (CRC) is a significant problem affecting patients all over the world. Since it is the fourth most common cause of cancer-related deaths, many scientists aim to expand their knowledge on the detection in early stages and treatment of this disease. Chemokines, as protein parameters involved in many processes accompanying the development of cancer, constitute a group of potential biomarkers that could also be useful in the detection of CRC. For this purpose, our research team used the results of thirteen parameters (nine chemokines, one chemokine receptor and three comparative markers, i.e., CEA, CA19-9 and CRP) to calculate one hundred and fifty indexes. Moreover, for the first time, the relationship between these parameters during the ongoing cancer process and in comparison to a control group are presented. As a result of statistical analyses using patients’ clinical data and the obtained indexes, it was established that several of the indexes have a diagnostic utility that is much higher than the tumor marker that is currently the most commonly used (CEA) currently. Furthermore, two of the indexes (CXCL14/CEA and CXCL16/CEA) showed not only extremely high usefulness in the detection of CRC in its early stages, but also the ability to determine whether the stage is low (stage I and II) or high (stage III and IV).

## 1. Introduction

Colorectal cancer (CRC) belongs to a group of neoplasms that constitute a significant socio-economic problem. Based on the number of cases, CRC is the fourth most common malignant tumor in the world. In recent years, a novel biomarker for the detection of early stages of CRC, KRAS and ctDNA/cfDNA, along with SEPT9 methylated DNA, has been established [1,2]. However, the molecular-biology methods used to detect these changes are still, to a certain extent difficult to access, especially in developing countries. Due to the fact that early-stage detection and screening for CRC are necessary, new, easy-to-perform and highly sensitive diagnostic methods are sought. A number of methods can be used in routine diagnosis to screen for and perform the identification of CRC in its early stages. Among others, fecal tests (e.g., FOBT and FIT) and colonoscopy, which is a relatively invasive method, are the most common methods for diagnosing CRC or confirming a positive result [1,3,4,5].

The increase in mortality due to CRC requires the use of screening tests, differential diagnostic procedures in cases in which a neoplastic lesion is detected, the implementation of treatment and the monitoring of the progress of the disease. This procedure is possible thanks to the use of biomarkers. Currently, the routinely used diagnostic markers include such parameters as carcinoembryonic antigen (CEA) and carbohydrate antigen 19-9 (CA 19-9). However, their usefulness is limited to monitoring the progress of the disease, especially after surgery, as their usefulness in detecting the development of the disease is very low. Therefore, new and more sensitive and specific biomarkers, with proven usefulness in the diagnosis of early-stage CRC, which would allow the use of more expensive and invasive methods to be avoided, are constantly being sought [3,6].

The identification of new and non-invasive biomarkers can be a useful screening method for CRC due to the simplicity of blood collection. A substantial number of non-hereditary cancers have genetic mutations in the early stages of carcinogenesis. Most of these abnormal cells from the growing tumor have the ability to secrete and their protein products can be found in biological materials, such as serum [7]. Therefore, we decided to extend the research conducted in our previous studies [8,9,10] (comprising the determination of chemokine concentrations in the course of colorectal cancer), with their calculated indexes. The aim of the use of these indexes was to observe not only changes in the concentrations of the tested parameters in the course of tumor development, but also the ratio of these changes between the tested parameters and the concentrations of routinely determined markers. This allows the observation of alterations in the dynamics, along with the changes in the number of cancer cells present in individual TNM-classification grades, with a simultaneous increase in the sensitivity and specificity of the test.

## 2. Materials and Methods

### 2.1. Materials

**Figure 1:**
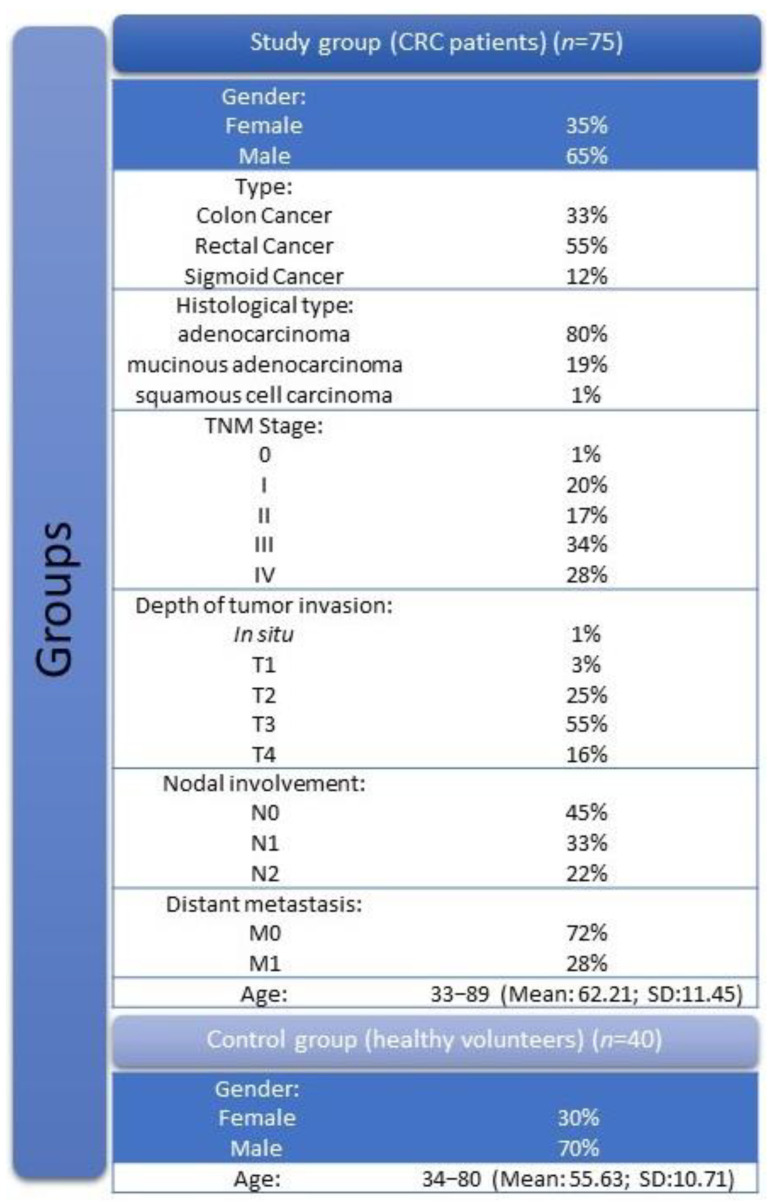
Characteristics of patients participating in the study.

This study was approved by the Bioethical Committee of the Medical University of Bialystok (no. R-I-002/564/2019). In this study, we used blood-serum samples from 115 patients (Figure 1), from whom informed consent was obtained. Whole group was divided into study group (*n* = 75) and control group (*n* = 40). All patients from study group suffered from colorectal cancer (CRC) and were diagnosed by the oncology group at Maria Sklodowska-Curie Oncology Center (Bialystok, Poland). Tumor classification and staging were conducted in accordance with the UICC-TNM classification. Colorectal cancer histopathology was based on the microscopic examination of tissue samples. The pretreatment staging procedures included physical and blood examinations, computed tomography (CT) and, in cases of patients with rectal cancer, magnetic resonance imaging (MRI) of the small pelvis. Additionally, all patients were assessed according to the Eastern Cooperative Oncology Group (ECOG) score. The blood was collected the day before the treatment (surgery, radio-, or chemotherapy). For each of the patients in the control group, the following exclusion criteria were applied: active infections and symptoms of an infection (both bacterial and viral), other comorbidities that can affect cytokine concentrations (respiratory diseases, digestive-tract diseases) or systemic diseases such as lupus, rheumatoid arthritis, or collagenosis. In addition, none of the patients included in the control group abused alcohol, smoked, or had a personal or familial history of cancer. None of the patients included (in both the study and control group) had a BMI > 35 kg/m^2^ to fully exclude the influence of an increase in obesity-related inflammatory factors.

### 2.2. Methods

The biochemical analysis of tested parameters was performed as described previously. Venous blood samples were collected from each patient into a tube with clot activator (S-Monovette, SARSTEDT, Nümbrecht, Germany), centrifuged to obtain serum samples and stored at −80 °C until assayed. The tested chemokines were measured with use of the Luminex 200 analyzer (multiplexing multiparametric fluorescence–laser reading system on microspheres for the simultaneous determination of multiple parameters) and Luminex Human Discovery Assay plates provided by R&D Systems, Abingdon, UK. According to the manufacturer’s protocols, duplicate samples were assessed for each standard, control and sample. Serum levels of classical tumor markers were measured with chemiluminescent microparticle immunoassay (CMIA) (Abbott, Chicago, IL, USA) and for the analysis of CRP concentration, immunoturbidymetric method (Abbott, Chicago, IL, USA) was used according to the manufacturer’s protocols [8,9,10]. As part of the planned study, 150 coefficients were calculated using the concentration results of the tested parameters (CCL11, CCL24, CCL26, CCR3, CCL2, CCL15, CCL4, CXCL16, CXCL5, CXCL14, CEA, CA19-9 and CRP). In total, 75 of these parameters were based on calculation of the index directly from the concentrations of all determined parameters (e.g., index A/B = the result of dividing the parameter A concentration by the parameter B concentration). A further 75 were based on calculation of indexes from the logarithms of concentrations to obtain a common unit of account (e.g., index log A/B = the result of dividing the logarithm of the parameter A concentration by the logarithm of the parameter B concentration).

Statistical analysis was performed by Statistica 13.0. Differences between two groups (study group—CRC patients and control group) were assessed using the Mann–Whitney U test. A Kruskal–Wallis rank ANOVA and post hoc tests were performed to show the differences between subgroups. The diagnostic performance of each test was calculated as SE, SP, PPV, NPV and ACC (diagnostic sensitivity; diagnostic specificity; positive/negative predictive values; and accuracy, respectively). We used the area under the ROC (receiver operating characteristics) curve to calculate the diagnostic performances of the tests. The cut-off points for each of the tested indexes were calculated with use of Youden’s index (Y-index). Only *p* values < 0.05 were considered as statistically significant.

## 3. Results

All 150 indexes were calculated using concentrations of CCL11, CCL24, CCL26, CCR3, CCL2, CCL15, CCL4, CXCL16, CXCL5, CXCL14, CEA, CA19-9 and CRP obtained in sera of 115 patients [8,9,10]. Since the patients’ results were not distributed normally, the non-parametric Mann–Whitney U test was performed to check the differences and their statistical significance between patients with CRC and healthy volunteers. All the results are summarized in Supplementary Table S1. 

Subsequently, for all the indexes whose *p* value on the Mann–Whitney U test remained <0.05, we conducted a further analysis. For this purpose, the cut-off point for each parameter was determined using the Youden’s index and the SE, SP, ACC, PPV, NPV and AUC values were calculated. Due to the amount and complexity of the data obtained, all values are presented in Table 1.

A further analysis was based on the performance of more than two subgroup comparisons using ANOVA and post hoc tests. All the statistically significant results concerning the differences between the indexes and cancer localization or advancement are presented in Table 2 and Table 3. Due to the small number of study participants diagnosed with TNM stages I and II of colorectal cancer, the division into individual stages I–IV was not statistically correct.

## 4. Discussion

Colorectal cancer (CRC) is known as the one of leading causes of death worldwide. Despite the progress made in the detection and treatment of this cancer, it still has a poor long-term prognosis. Furthermore, serious challenges persist, due to late diagnosis and non-successful treatment [3]. Biomarkers allowing the detection of ongoing changes in the body at early stages are key tools for rapid detection, prognoses, increasing patient survival and predicting responses to treatment. In recent years, significant progress has been made in assessing the usefulness of cancer biomarkers, which has allowed greater individualization of therapy, with a positive impact on survival outcomes [11]. However, there are still no parameters that clearly indicate ongoing pathological processes and that are, at the same time, broadly accessible, simple and quick to perform, without posing a risk to the patient (non-invasive methods). Therefore, in our work, we tried to develop the most sensitive and specific diagnostic test that would allow the determination of these parameters in easily accessible biological material (blood serum) using rapid and widely available assays.

Chemokines, the concentrations of which were determined in the blood sera of patients and were used to calculate indexes, belong to the group of chemotactic cytokines. These are a family of low-molecular-mass proteins that play an important role in many processes, both physiological and pathological. Chemokines take part in the development of inflammatory processes (during cancer progression or even infection with, e.g., COVID-19). Through connections with chemokine receptors, they have a chemotactic effect and influence the migration of immune-system cells in tissues. The effects of chemokines have been found in: the stimulation and migration of various leukocyte populations, anti-infectious immunity, hemo- and lymphopoiesis, embryogenesis, organogenesis, the regulation of apoptosis and angiogenesis. Some of these processes are significantly involved in cancer progression and metastasis, which encouraged our research team to focus on the role of these proteins in the development of colorectal cancer, to investigate how their concentrations change in the course of this disease and to determine whether there are dependencies between their concentrations [12,13,14,15,16,17].

The median concentrations of most of the parameters studied by our research team were higher in the sera of the CRC patients (CCL2, CCL4, CCL15, CCL24, CCR3, CXCL5, CXCL16, CEA and CRP), but not all of these differences were statistically significant. The concentrations of other parameters (CCL11, CCL26, CXCL14 and CA 19-9) were comparable to or even lower in the sera of patients with colorectal cancer compared to the group of healthy volunteers [8,9,10]. Similar results were obtained by other researchers not only for the concentrations of the parameters studied, but also for their expression, which prompted us to conduct further analyses [18,19,20,21,22,23,24,25,26,27,28,29].

Summarizing the obtained results, we decided to calculate the indexes from all the obtained concentrations and check whether the relationships between all the previously mentioned parameters could be used as CRC biomarkers. Interestingly, after calculating the indexes and the performances on the Mann–Whitney U test, we obtained very interesting results, indicating the usefulness of 57 out of the 150 calculated indexes through the differentiation between the cancer and the control groups (Table 1). It should be noted that these were the first analyses to focus on the calculation of the indexes of the tested chemokines in the course of CRC, aiming to show the relationship between alterations in the concentrations of individual parameters. Similar papers, calculating indexes in the course of other diseases and parameters, were published previously. Some have also been introduced into routine diagnostics [30,31,32,33,34].

As a result of our attempt to determine the diagnostic usefulness of these parameters, we conducted further analyses (Table 2). The AUC values of all the indexes exceeded the AUC value previously obtained for the routine marker, CA 19-9 [8,9,10]. None of the AUC values were lower than 0.610, which indicates high potential usefulness compared to the AUC values obtained for the single parameters tested (the highest AUC was that of CCR3, 0.683) [8]. 

Some of the studied indexes had an AUC higher than the CEA (CCL11/CRP, CCL26/CRP, CCL2/CRP, CCL15/CRP, CCL4/CRP, CXCL16/CRP, CXCL16/CEA, CXCL14/CRP, CXCL16/CEA). These indexes were calculated directly on the basis of the chemokine concentrations, not their logarithms, which indicates that the introduction of a common unit for calculations may not be as significant as we had assumed. Interestingly, all of the aforementioned indexes concerned one of the chemokines and a routine marker (CEA) or an inflammatory marker (CRP). The CEA, which was discovered as a fetal-derived glycoprotein, is detected in the normal tissues of the intestines, pancreas and livers of fetuses. Since 1965, it has been a useful tumor marker for adenocarcinomas, i.e., tumors defined as neoplasia of epithelial tissues with a glandular origin, such as CRC [35,36]. This may be said to prove the high level of usefulness of the indexes described above in CRC diagnostics, as the presence of CEA in particular is highly specific to GI (gastrointestinal) tumors and CRC itself. 

Of the aforementioned indexes, the highest AUC values and, at the same time, the highest level of diagnostic usefulness, were demonstrated by the CCL26/CRP, CXCL14/CRP and CXCL16/CRP, reaching AUC values above 0.850. The sensitivity (SE), which is the ability of a test to detect the certain condition [37], of the tested parameters reached values up to 95%, which are extremely high values compared to the previously obtained values of individual parameters (up to 80%) [8,9,10]. The diagnostic specificities of the indexes (SP, which measures the ability of a test to detect the absence of a disease) reached values up to 82.61%, confirming their high level of usefulness. In our opinion, reaching higher levels of SP would have been difficult as both CRP and chemokines have low organ specificity. The PPV and NPV values (the probability of correctly identifying the disease) reached 75% and 96.08%, respectively, and were much higher than previous values [8,9,10]. The ACC, which is the likelihood of the test correctly differentiating between patients and healthy volunteers [37], had not been applied previously, but for the examined indexes, it was as high as 79.49%, in the case of the CCL26/CRP index. 

Later in the analysis, ANOVA and post hoc tests were performed. Interestingly, we did not observe any statistically significant differences between colon and rectal cancer, which proves the occurrence of similar changes in the parameters studied in both types of cancer. However, we observed changes between these two types of CRC and sigmoid carcinoma. The observed differences are presented in Table 3, and the resulting statistically significant differences may have been related not only to different parts of the intestine in which neoplastic changes were observed, but also to the fact that all the patients with sigmoid cancer in our study were in the advanced stages (III and IV) of the TNM classification, in which both local and distant metastases are observed. This fact might have affected the obtained results and is considered as the biggest flaw of this study.

Our last analysis concerned the observation of differences in the studied indexes between the stages of cancer advancement. Due to the small number of cases in stage I and II, we divided them into a control group, comprising patients with low levels of cancer development (without metastases, TNM stages I and II) and an advanced-cancer group (with local and distant metastases, TNM stages III and IV). As a result of this analysis, we obtained significant data on the alterations in the indexes depending on the degree of advancement. For indexes such as CCL11/CA 19-9, log CCL11/CA 19-9, log CCL24/CA 19-9 and CCR3/CA 19-9, we observed statistically significant differences only when comparing the control group and the group with low levels of cancer development (locally limited), which may indicate the possibility of the detection of early-stages changes in the course of neoplastic disease. For the indexes CCL26/CCR3, CXCL5/CEA, log CXCL14/CA 19-9, CCL26/CCL2, log CCL26/CCL2, log CCR3/CCL2, CCL15/CXCL5, CCL4/CXCL16, CCL4/CXCL14, log CCL4/CXCL14, CXCL16/CXCL5, log CXCL16/CXCL5, CXCL5/CXCL14 and log CXCL5/CXCL14, we observed statistically significant differences only in the comparison between the control group and the group with advanced cancer, which proves their relationship with the presence of metastases. The production of these indexes may be closely related to increased angiogenesis.

For the indexes log CCL11/CCR3, log CCL24/CCR3, CCL11/CRP, CCL24/CRP, CCL26/CEA, CCL26/CRP, CCR3/CRP, CCL2/CRP, CCL15/CRP, CCL4/CRP, CXCL16/CRP, CXCL5 /CRP, CXCL14/CRP, CCR3/CXCL14, CCL2/CXCL14 and log CCL2/CXCL14, we observed statistically significant differences in the comparison between the control group and those with both stages of cancer advancement, but we did not observe significant differences between the low and high levels of advancement. These results indicate the occurrence of alterations in the examined parameters appear already at the beginning of cancer, which are highly useful in detecting the disease, but cannot be used to assess the advancement of neoplastic changes.

The indexes that showed differentiating values between the stages of advancement, but did not show differences between the cancer and control groups, were as follows: index CCL15/CA 19-9, log CCL15/CA 19-9, CCL4/CA 19-9, log CCL4/CA 19-9, CXCL16/CA 19-9, log CXCL16/CA 19-9, log CXCL5/CA 19-9, CCL2/CCL4, log CCL2/CCL4 and CCL2/CXCL5. On the other hand, the indexes which, apart from the differences between the stages of advancement, also showed statistically significant differences between the controls and those with low or high degree of advancement, were as follows: index CCL2/CA 19-9, log CCL2/CA 19-9, log CCL2/CEA, CCL15/CEA, log CCL4/CEA, CXCL14/CA 19-9, log CCL15/CXCL5 and log CCL4/CXCL16. These parameters and the differences between their values indicate processes occurring in the course of neoplastic formation, but their use is not conducive to the detection of neoplastic lesions.

Most importantly, the CXCL16/CEA and CXCL14/CEA indexes showed statistically significant differences in all cases, which could suggest that they offer the highest degree of usefulness not only in the detection, but also in the determination of the stage of cancer, even before the histopathological examination, which is the most important result of the research conducted. Conceivably, the introduction of these tests to routine screening diagnostics would make it possible to increase the detection of CRC worldwide. However, this requires further research and confirmation. Interestingly, Abdel Mageed M et al. [28] revealed that the mRNA expressions of CXCL14 and CXCL16 in colon cancer tissues were statistically higher compared to normal colon tissues. However, of the cell lines studied, only CXCL16 showed similar results. Interestingly, the authors also did not show a relationship between the mRNA of the studied chemokines and the stage of cancer advancement. This discrepancy might be connected with the small number of samples tested. In addition the authors discovered significant differences between the cancerous lymph nodes and the control nodes only in terms of CXCL16. The authors concluded that ‘CXCL16 mRNA is a marker for poor prognosis both independently or in combination with CEA mRNA and that it merits further studies.’ Although they were derived using completely different research methods and by setting different goals during the study design, these results partially coincide with our conclusions, as CXCL16 can be considered as an important parameter that can be useful in the detection, staging and prognosis of patients with CRC.

Our research group considers that the indexes with the highest degree of usefulness as potential routine markers of CRC, CXCL16/CEA and CXCL14/CEA, are not without significance. We believe that the CEA marker used so far shows quite high specificity, but lacks the high sensitivity needed to classify patients as sick or healthy, which is ensured by the determination of the concentration of fast-reacting pro-inflammatory chemokines. Perhaps the basic markers discovered so far only require the addition of a second parameter to increase their usefulness. We hope that this research will encourage other scientists to establish whether the determination of chemokines in other cancers and the creation of similar indexes with adequate basic markers (e.g., CA 15-3 in breast cancer or CA 72-4 in gastric cancer) makes it possible to obtain similar results.

## 5. Conclusions

Currently, many researchers are searching for biomarkers that would allow the rapid identification of ongoing neoplastic processes. In this work, our scientific team focused on the calculation of one hundred and fifty indexes from the determined concentrations of ten chemokine-family proteins and three comparative markers. As a result of the analyses conducted, it was possible to determine that several of the indexes indexes could be used in the diagnosis of early-stage CRC, as biomarkers of this cancer. These parameters include, first of all, the CXCL14/CEA and CXCL16/CEA indexes, which showed extremely high diagnostic usefulness and the possibility of assessing and differentiating between CRC and healthy volunteers, as well as between the stages of cancer advancement. Therefore, their introduction to screening diagnostics, after the obtained results are confirmed, could be beneficial.

## Figures and Tables

**Table 1:** Diagnostic utility of all statistically significant indexes.

Parameter	Cut-Off	AUC	*p*	SE	SP	ACC	PPV	NPV	Y-Index
index log CCL11/CCR3	−1.491	0.738	<0.001	87.10%	56.52%	68.83%	57.45%	86.67%	0.436
index log CCL24/CCR3	−4.817	0.734	<0.001	96.77%	45.65%	66.23%	54.55%	95.45%	0.424
index CCL26/CCR3	140.833	0.696	0.001	59.38%	76.60%	69.62%	63.33%	73.47%	0.360
index log CCL26/CCR3	−1.629	0.652	0.014	68.75%	61.70%	64.56%	55.00%	74.36%	0.305
index CCL11/CEA	8.176	0.641	0.029	67.74%	56.52%	61.04%	51.22%	72.22%	0.243
index log CCL11/CEA	−3.854	0.656	0.024	91.30%	44.83%	73.30%	72.41%	76.47%	0.361
index CCL11/CA 19-9	1.571	0.647	0.025	78.26%	51.61%	67.53%	70.59%	61.54%	0.299
index log CCL11/CA 19-9	1.934	0.655	0.018	50.00%	80.65%	62.34%	79.31%	52.08%	0.306
index CCL11/CRP	5.652	0.797	<0.001	80.65%	69.57%	74.03%	64.10%	84.21%	0.502
index CCL24/CEA	1125.435	0.635	0.037	54.84%	68.89%	63.16%	54.84%	68.89%	0.237
index log CCL24/CEA	−7.600	0.644	0.036	84.44%	51.72%	71.62%	73.08%	68.18%	0.362
index CCL24/CA 19-9	367.340	0.638	0.034	51.11%	80.65%	63.16%	79.31%	53.19%	0.318
index log CCL24/CA 19-9	3.472	0.635	0.041	82.22%	48.39%	68.42%	69.81%	65.22%	0.306
index CCL24/CRP	694.554	0.772	<0.001	74.19%	73.33%	73.68%	65.71%	80.49%	0.475
index CCL26/CEA	21.600	0.728	<0.001	59.38%	82.61%	73.08%	70.37%	74.51%	0.420
index log CCL26/CEA	0.476	0.651	0.027	82.61%	53.33%	71.05%	73.08%	66.67%	0.359
index CCL26/CRP	10.476	0.858	<0.001	75.00%	82.61%	79.49%	75.00%	82.61%	0.576
index CCR3/CEA	0.120	0.623	0.040	58.97%	67.35%	63.64%	58.97%	67.35%	0.263
index CCR3/CA 19-9	0.023	0.634	0.026	83.67%	46.15%	67.05%	66.13%	69.23%	0.298
index CCR3/CRP	0.035	0.768	<0.001	94.87%	51.02%	70.45%	60.66%	92.59%	0.459
index CCL2/CEA	280.587	0.718	<0.001	67.50%	73.33%	71.30%	57.45%	80.88%	0.408
index CCL2/CRP	159.228	0.797	<0.001	80.00%	68.00%	72.17%	57.14%	86.44%	0.480
index CCL15/CEA	1049.790	0.742	<0.001	75.00%	65.33%	68.70%	53.57%	83.05%	0.403
index CCL15/CRP	893.125	0.800	<0.001	77.50%	69.33%	72.17%	57.41%	85.25%	0.468
index CCL4/CEA	167.160	0.740	<0.001	67.50%	72.00%	70.43%	56.25%	80.60%	0.395
index CCL4/CRP	121.474	0.800	<0.001	77.50%	74.67%	75.65%	62.00%	86.15%	0.522
index CXCL16/CEA	812.022	0.804	<0.001	60.00%	88.00%	78.26%	72.73%	80.49%	0.480
index CXCL16/CRP	247.800	0.859	<0.001	92.50%	66.67%	75.65%	59.68%	94.34%	0.592
index CXCL5/CEA	513.910	0.731	<0.001	72.50%	66.67%	68.70%	53.70%	81.97%	0.392
index CXCL5/CRP	379.917	0.775	<0.001	77.50%	70.67%	73.04%	58.49%	85.48%	0.482
index CXCL14/CEA	277.530	0.816	<0.001	90.00%	65.33%	73.91%	58.06%	92.45%	0.553
index CXCL14/CRP	161.435	0.877	<0.001	95.00%	65.33%	75.65%	59.38%	96.08%	0.603
index CCL11/ CCL26	0.450	0.646	0.025	91.11%	35.48%	68.42%	67.21%	73.33%	0.266
index log CCL11/ CCL26	0.741	0.644	0.027	91.11%	35.48%	68.42%	67.21%	73.33%	0.266
index CCL11/CXCL16	0.010	0.647	0.025	93.48%	29.03%	67.53%	66.15%	75.00%	0.225
index CCL11/CXCL14	0.019	0.649	0.022	69.57%	58.06%	64.94%	71.11%	56.25%	0.276
index log CCL11/CXCL14	0.346	0.648	0.023	93.48%	38.71%	71.43%	69.35%	80.00%	0.322
index CCL26/CCL2	0.055	0.675	0.005	62.50%	71.74%	67.95%	60.61%	73.33%	0.342
index log CCL26/CCL2	0.511	0.662	0.012	65.63%	65.22%	65.38%	56.76%	73.17%	0.308
index log CCR3/CCL2	−0.304	0.669	0.004	69.39%	64.10%	67.05%	70.83%	62.50%	0.335
index log CCR3/CCL15	−0.226	0.627	0.035	63.27%	61.54%	62.50%	67.39%	57.14%	0.248
index CCL2/CXCL14	0.848	0.695	<0.001	52.00%	82.50%	62.61%	84.78%	47.83%	0.345
index log CCL2/CXCL14	0.975	0.697	<0.001	52.00%	82.50%	62.61%	84.78%	47.83%	0.345
index CCL15/CXCL5	1.272	0.611	0.040	92.50%	34.67%	54.78%	43.02%	89.66%	0.272
index log CCL15/CXCL5	1.041	0.615	0.035	90.00%	34.67%	53.91%	42.35%	86.67%	0.247
index CCL4/CXCL16	0.335	0.624	0.035	72.00%	55.00%	66.09%	75.00%	51.16%	0.270
index log CCL4/CXCL16	0.836	0.624	0.036	76.00%	52.50%	67.83%	75.00%	53.85%	0.285
index CCL4/CXCL14	0.465	0.691	0.001	74.67%	60.00%	69.57%	77.78%	55.81%	0.347
index log CCL4/CXCL14	0.845	0.691	0.001	86.67%	47.50%	73.04%	75.58%	65.52%	0.342
index CXCL16/CXCL5	0.802	0.642	0.006	67.50%	56.00%	60.00%	45.00%	76.36%	0.235
index log CXCL16/CXCL5	0.959	0.644	0.006	75.00%	50.67%	59.13%	44.78%	79.17%	0.257
index CXCL16/CXCL14	1.593	0.618	0.027	46.67%	80.00%	58.26%	81.40%	44.44%	0.267
index log CXCL16/CXCL14	1.071	0.619	0.026	49.33%	80.00%	60.00%	82.22%	45.71%	0.293
index CXCL5/CXCL14	2.832	0.687	<0.001	42.67%	90.00%	59.13%	88.89%	45.57%	0.327
index log CXCL5/CXCL14	1.060	0.688	<0.001	69.33%	62.50%	66.96%	77.61%	52.08%	0.318

*p*—statistical significance of AUC (comparison to AUC = 0.5, which is the borderline of the diagnostic usefulness of the test); SE—sensitivity; SP—specificity; ACC—accuracy; PPV—positive predictive value; NPV—negative predictive value; Y-index—Youden’s index.

**Table 2:** ANOVA and post hoc analysis results for different cancer localizations.

Parameter	ANOVA	Post-Hoc		
	*p*	Colon vs. Rectal	Colon vs. Sigmoid	Rectal vs. Sigmoid
index CCL2/CEA	0.006	N/S	0.029	0.004
index CCL15/CEA	0.003	N/S	N/S	0.003
index CCL4/CEA	0.004	N/S	0.025	0.002
index CXCL16/CEA	0.002	N/S	0.025	0.001
index CXCL5/CEA	0.007	N/S	0.013	0.007
index CXCL14/CEA	0.002	N/S	0.016	0.001
index CCR3/CCL2	0.029	N/S	N/S	0.039

*p*—statistical significance assessed by the Kruskal–Wallis ANOVA test; N/S—non-significant result.

**Table 3:** ANOVA and post hoc analysis results for stages of different cancer advancement.

Parameter	*p*	C vs. I + II	C vs. III + IV	I + II vs. III + IV
index log CCL11/CCR3	0.002	0.019	0.003	N/S
index log CCL24/CCR3	0.001	0.045	0.001	N/S
index CCL26/CCR3	0.012	N/S	0.024	N/S
index CCL11/CA 19-9	0.023	0.020	N/S	N/S
index log CCL11/CA 19-9	0.021	0.017	N/S	N/S
index CCL11/CRP	<0.001	<0.001	0.007	N/S
index log CCL24/CA 19-9	0.041	0.038	N/S	N/S
index CCL24/CRP	<0.001	<0.001	0.040	N/S
index CCL26/CEA	0.002	0.029	0.004	N/S
index CCL26/CRP	<0.001	<0.001	<0.001	N/S
index CCR3/CA 19-9	0.042	0.035	N/S	N/S
index CCR3/CRP	<0.001	<0.001	0.008	N/S
index CCL2/CEA	<0.001	N/S	<0.001	0.004
index CCL2/CA 19-9	0.006	0.031	N/S	0.007
index log CCL2/CA 19-9	0.001	0.047	N/S	0.001
index CCL2/CRP	<0.001	<0.001	<0.001	N/S
index CCL15/CEA	<0.001	N/S	<0.001	0.002
index CCL15/CA 19-9	0.009	N/S	N/S	0.008
index log CCL15/CA 19-9	0.002	N/S	N/S	0.002
index CCL15/CRP	<0.001	<0.001	<0.001	N/S
index CCL4/CEA	<0.001	N/S	<0.001	0.011
index CCL4/CA 19-9	0.038	N/S	N/S	0.046
index log CCL4/CA 19-9	0.002	N/S	N/S	0.001
index CCL4/CRP	<0.001	<0.001	<0.001	N/S
index CXCL16/CEA	<0.001	0.048	<0.001	0.005
index CXCL16/CA 19-9	0.006	N/S	N/S	0.004
index log CXCL16/CA 19-9	0.001	N/S	N/S	0.001
index CXCL16/CRP	<0.001	<0.001	<0.001	N/S
index CXCL5/CEA	<0.001	N/S	<0.001	N/S
index log CXCL5/CA 19-9	0.003	N/S	N/S	0.002
index CXCL5/CRP	<0.001	<0.001	0.001	N/S
index CXCL14/CEA	<0.001	0.027	<0.001	0.006
index CXCL14/CA 19-9	0.001	N/S	0.025	0.002
index log CXCL14/CA 19-9	0.001	N/S	<0.001	N/S
index CXCL14/CRP	<0.001	<0.001	<0.001	N/S
index CCL26/CCL2	0.007	N/S	0.005	N/S
index log CCL26/CCL2	0.022	N/S	0.019	N/S
index log CCR3/CCL2	0.017	N/S	0.020	N/S
index CCR3/CXCL14	0.002	0.002	0.049	N/S
index CCL2/CCL4	0.027	N/S	N/S	0.024
index log CCL2/CCL4	0.021	N/S	N/S	0.020
index CCL2/CXCL5	0.048	N/S	N/S	0.042
index CCL2/CXCL14	0.003	0.020	0.005	N/S
index log CCL2/CXCL14	0.003	0.021	0.004	N/S
index CCL15/CXCL5	0.009	N/S	0.017	N/S
index log CCL15/CXCL5	0.007	N/S	0.013	0.049
index CCL4/CXCL16	0.009	N/S	0.012	N/S
index log CCL4/CXCL16	0.004	N/S	0.007	0.033
index CCL4/CXCL14	<0.001	N/S	<0.001	N/S
index log CCL4/CXCL14	<0.001	N/S	<0.001	N/S
index CXCL16/CXCL5	0.005	N/S	0.005	N/S
index log CXCL16/CXCL5	0.005	N/S	0.005	N/S
index CXCL5/CXCL14	<0.001	N/S	<0.001	N/S
index log CXCL5/CXCL14	<0.001	N/S	<0.001	N/S

*p*—statistical significance assessed by the Kruskal–Wallis ANOVA test; C—control group; I–IV—tumor TNM stage; N/S—non-significant result.

## Data Availability

The data presented in this study are available on request from the corresponding author. Key data are stated in the text.

## Supplementary Materials

The following supporting information can be downloaded at: https://www.mdpi.com/article/10.3390/jcm12103530/s1, Table S1. Statistical differences between study (colorectal cancer) and control groups.
