# Supplementary material for: A Novel Approach to Staging and Detection of Colorectal Cancer in Early Stages
Source: J Clin Med. 2023 May 17;12(10):3530. doi: 10.3390/jcm12103530 (PMC10218832; doi:10.3390/jcm12103530)
Supplement: Supplementary file 1 [file jcm-12-03530-s001.zip › jcm-2348267-supplementary.pdf]

Table S1. Statistical differences between Study (colorectal cancer) and Control groups.

Parameter	*p*	Parameter	*p*	Parameter	*p*
index CCL11/CCR3	0.691	index CXCL16/CA 19-9	0.836	index CCL26/CCL4	0.080
index log CCL11/CCR3	<0.001	index log CXCL16/CA 19-9	0.877	index log CCL26/CCL4	0.117
index CCL24/CCR3	0.683	index CXCL16/CRP	<0.001	index CCL26/CXCL16	0.948
index log CCL24/CCR3	<0.001	index log CXCL16/CRP	0.224	index log CCL26/CXCL16	0.876
index CCL26/CCR3	0.003	index CXCL5/CEA	<0.001	index CCL26/CXCL5	0.393
index log CCL26/CCR3	0.022	index log CXCL5/CEA	0.177	index log CCL26/CXCL5	0.387
index CCL11/CEA	0.037	index CXCL5/CA 19-9	0.369	index CCL26/CXCL14	0.295
index log CCL11/CEA	0.023	index log CXCL5/CA 19-9	0.863	index log CCL26/CXCL14	0.399
index CCL11/CA 19-9	0.030	index CXCL5/CRP	<0.001	index CCR3/CCL2	0.336
index log CCL11/CA 19-9	0.021	index log CXCL5/CRP	0.319	index log CCR3/CCL2	0.007
index CCL11/CRP	<0.001	index CXCL14/CEA	<0.001	index CCR3/CCL15	0.327
index log CCL11/CRP	0.717	index log CXCL14/CEA	0.231	index log CCR3/CCL15	0.042
index CCL24/CEA	0.046	index CXCL14/CA 19-9	0.196	index CCR3/CCL4	0.251
index log CCL24/CEA	0.037	index log CXCL14/CA 19-9	0.634	index log CCR3/CCL4	0.140
index CCL24/CA 19-9	0.041	index CXCL14/CRP	<0.001	index CCR3/CXCL16	0.009
index log CCL24/CA 19-9	0.046	index log CXCL14/CRP	0.164	index log CCR3/CXCL16	0.159
index CCL24/CRP	<0.001	index CCL11/ CCL24	0.950	index CCR3/CXCL5	0.581
index log CCL24/CRP	0.629	index log CCL11/ CCL24	0.214	index log CCR3/CXCL5	0.055
index CCL26/CEA	0.001	index CCL11/ CCL26	0.031	index CCR3/CXCL14	<0.001
index log CCL26/CEA	0.026	index log CCL11/ CCL26	0.032	index log CCR3/CXCL14	0.280
index CCL26/CA 19-9	0.196	index CCL11/ CCL2	0.979	index CCL2/ CCL15	0.187
index log CCL26/CA 19-9	0.101	index log CCL11/ CCL2	0.366	index log CCL2/ CCL15	0.117
index CCL26/CRP	<0.001	index CCL11/ CCL15	0.094	index CCL2/ CCL4	0.667
index log CCL26/CRP	0.421	index log CCL11/ CCL15	0.067	index log CCL2/ CCL4	0.549
index CCR3/CEA	0.048	index CCL11/ CCL4	0.865	index CCL2/CXCL16	0.105
index log CCR3/CEA	0.125	index log CCL11/ CCL4	0.399	index log CCL2/CXCL16	0.087
index CCR3/CA 19-9	0.030	index CCL11/CXCL16	0.029	index CCL2/CXCL5	0.881
index log CCR3/CA 19-9	0.646	index log CCL11/CXCL16	0.068	index log CCL2/CXCL5	0.858
index CCR3/CRP	<0.001	index CCL11/CXCL5	0.123	index CCL2/CXCL14	0.001
index log CCR3/CRP	0.186	index log CCL11/CXCL5	0.090	index log CCL2/CXCL14	<0.001
index CCL2/CEA	<0.001	index CCL11/CXCL14	0.028	index CCL15/ CCL4	0.255
index log CCL2/CEA	0.179	index log CCL11/CXCL14	0.030	index log CCL15/ CCL4	0.196
index CCLl2/CA 19-9	0.360	index CCL24/CCL26	0.163	index CCL15/CXCL16	0.909
index log CCL2/CA 19-9	0.795	index log CCL24/CCL26	0.109	index log CCL15/CXCL16	0.923
index CCL2/CRP	<0.001	index CCL24/CCL2	0.638	index CCL15/CXCL5	0.050
index log CCL2/CRP	0.249	index log CCL24/CCL2	0.602	index log CCL15/CXCL5	0.043
index CCL15/CEA	<0.001	index CCL24/CCL15	0.266	index CCL15/CXCL5	0.378
index log CCL15/CEA	0.208	index log CCL24/15	0.266	index log CCL15/CXCL5	0.327
index CCL15/CA 19-9	0.663	index CCL24/4	0.849	index CCL4/CXCL16	0.028
index log CCL15/CA 19-9	0.663	index log CCL24/4	0.988	index log CCL4/CXCL16	0.030
index CCL15/CRP	<0.001	index CCL24/CXCL16	0.294	index CCL4/CXCL5	0.813
index log CCL15/CRP	0.238	index log CCL24/CXCL16	0.298	index log CCL4/CXCL5	0.719
index CCL4/CEA	<0.001	index CCL24/CXCL5	0.328	index CCL4/CXCL14	0.001
index log CCL4/CEA	0.206	index log CCL24/CXCL5	0.355	index log CCL4/CXCL14	0.001
index CCL4/CA 19-9	0.418	index CCL24/CXCL14	0.212	index CXCL16/CXCL5	0.012
index log CCL4/CA 19-9	0.759	index log CCL24/CXCL14	0.194	index log CXCL16/CXCL5	0.011
index CCL4/CRP	<0.001	index CCL26/CCL2	0.008	index CXCL16/CXCL14	0.038
index log CCL4/CRP	0.264	index log CCL26/CCL2	0.015	index log CXCL16/CXCL14	0.035
index CXCL16/CEA	<0.001	index CCL26/CCL15	0.589	index CXCL5/CXCL14	0.001
index log CXCL16/CEA	0.220	index log CCL26/CCL15	0.820	index log CXCL5/CXCL14	0.001

p—statistical significance assessed by the Mann-Whitney U test.
